# Disparities in Thyroid Cancer Diagnosis Based on Residence and Distance From Medical Facility

**DOI:** 10.1210/jendso/bvae033

**Published:** 2024-02-18

**Authors:** Sunita Regmi, Paraskevi A Farazi, Elizabeth Lyden, Anupam Kotwal, Apar Kishor Ganti, Whitney Goldner

**Affiliations:** Kansas Department of Health and Environment, Bureau of Epidemiology and Public Health Informatics, Curtis State Office Building, 1000 SW Jackson ST., Suite 130, Topeka, KS 66612-1365, USA; Department of Epidemiology, University of Nebraska Medical Center, College of Public Health, Omaha, NE 68198, USA; Department of Epidemiology, University of Nebraska Medical Center, College of Public Health, Omaha, NE 68198, USA; School of Medicine, European University Cyprus, 6 Diogenous Street, 2404 Engomi, Nicosia, Cyprus; Department of Biostatistics, University of Nebraska Medical Center, College of Public Health, Omaha, NE 68198, USA; Department of Medicine, Division of Diabetes, Endocrinology and Metabolism, University of Nebraska Medical Center, Omaha, NE 68198, USA; Department of Medicine, Division of Hematology and Oncology, University of Nebraska Medical Center, Omaha, NE 68198, USA; VA Nebraska Western Iowa Health Care System, 4101 Woolworth Avenue, Omaha, NE 68105-1850, USA; Department of Medicine, Division of Diabetes, Endocrinology and Metabolism, University of Nebraska Medical Center, Omaha, NE 68198, USA

**Keywords:** thyroid cancer, disparities, access to care, treatment, presentation

## Abstract

**Context:**

Rural-urban disparities have been reported in cancer care, but data are sparse on the effect of geography and location of residence on access to care in thyroid cancer.

**Objective:**

To identify impact of rural or urban residence and distance from treatment center on thyroid cancer stage at diagnosis.

**Methods:**

We evaluated 800 adults with differentiated thyroid cancer in the iCaRe2 bioinformatics/biospecimen registry at the Fred and Pamela Buffett Cancer Center. Participants were categorized into early and late stage using AJCC staging, and residence/distance from treating facility was categorized as short (≤ 12.5 miles), intermediate (> 12.5 to < 50 miles) or long (≥ 50 miles). Multivariable logistic regression was used to identify factors associated with late-stage diagnosis.

**Results:**

Overall, 71% lived in an urban area and 29% lived in a rural area. Distance from home to the treating facility was short for 224 (28%), intermediate for 231 (28.8%), and long for 345 (43.1%). All 224 (100%) short, 226 (97.8%) intermediate, and 120 (34.7%) long distances were for urban patients; in contrast, among rural patients, 5 (2.16%) lived intermediate and 225 (65.2%) lived long distances from treatment (*P* < .0001). Using eighth edition AJCC staging, the odds ratio of late stage at diagnosis for rural participants ≥ 55 years was 2.56 (95% CI, 1.08-6.14) (*P* = .03), and for those living ≥ 50 miles was 4.65 (95% CI, 1.28-16.93) (*P* = .0075). Results were similar using seventh edition AJCC staging.

**Conclusion:**

Older age at diagnosis, living in rural areas, and residing farther from the treatment center are all independently associated with late stage at diagnosis of thyroid cancer.

Thyroid cancer, the most common endocrine malignancy, accounts for 2.2% of newly diagnosed cancers in the United States, with an estimated 43 720 new cases in 2023 [1]. Thyroid cancer incidence tripled from 1995 to 2008, in part due to increased detection, but most cases were small papillary thyroid carcinomas and the incidence trend plateaued between 2010 and 2019 [2-4]. The incidence of thyroid cancer varies with age, gender, and race/ethnicity [5]. Hispanic females, age 20 to 49 years, had an increase in thyroid cancer incidence without deceleration after 2009, whereas elderly non-Hispanic White females had a decrease in incidence but stable mortality rate [6]. According to the Nebraska Cancer Registry, between 2015 and 2019, 37% of thyroid cancer cases were aged 18 to 44 years, 42% were 45 to 64 years, and 21% were 65 years and older. The Nebraska incidence rate during 2015 to 2019 was higher at 15.8 per 100 000 compared with 14.1 per 100 000 for the overall US population from 2014 to 2018 [7]. Female individuals are 3 times more likely to be diagnosed with thyroid cancer, with the highest rates among Asian and Pacific Islander and Hispanic female populations compared to other races [8, 9].

In the United States, about 90% of thyroid cancers are considered differentiated thyroid cancer (DTC) including papillary, follicular, and oncocytic, previously termed Hurthle cell [10]. Male sex, increased age, lymph node metastasis, large tumor size, and histologically undifferentiated cancer are associated with an adverse outcome [11], and staging at diagnosis is a strong predictor of survival [1, 12]. The American Thyroid Association's Risk of Recurrence stratification (ROR) is a more appropriate predictor of tumor behavior, persistence, and recurrence [13, 14]. The American Joint Committee on Cancer (AJCC) TNM Staging defines thyroid cancer based on TNM (extent of the tumor-T, spread to nearby lymph nodes-N, and to distant metastasis-M) and overall stage (I-IV). It also considers age at diagnosis, categorizing as stage I all patients < 45 years of age at the time of diagnosis in the absence of distant metastasis for the seventh edition and < 55 years at the time of diagnosis for the eighth edition. Patients diagnosed at ≥ 45 years or ≥ 55 years as per the seventh and eighth edition are considered higher stage than the same TNM counterparts who are < 45 or < 55 years, respectively.

Disparities exist across the entire spectrum of cancer care, including thyroid cancer. Although these disparities are multifactorial, studies attribute race, geography, health care access, and education level as significant factors for inequities in cancer care. Distance from the treatment center, lower socioeconomic status, and non-private insurance or being uninsured are associated with delays in diagnosis and treatment [5, 15, 16]. There are numerous examples from many states that have reported persistence of environmental toxins, endocrine disruptors, radioactive material, and waste in landfills that persist for significant periods of time exposing persons living near these areas to these toxins and pollutants in the soil or drinking water, even once the areas have been repurposed [17, 18].

Studies have also shown a wider gap in the stage at diagnosis not only across racial/ethnic minorities but also in medically underserved populations, including those living in rural areas. Although the cancer incidence rate declined in metropolitan and rural areas, mortality rate declined less in the rural populations [16, 19]. Several studies recently have shown that underserved populations are less likely to receive the standard of care recommended for the type and stage of cancer, which suggests substantial barriers to health care access among rural residents (underserved population) that need further research [20].

Henley et al evaluated cancer incidence and mortality using the Centers for Disease Control (CDC)'s National Program of Cancer Registries and National Cancer Institute (NCI)'s Surveillance, Epidemiology, and End Results (SEER) database and found nonmetropolitan (rural) areas to have higher incidence and death rates compared to metropolitan areas for many different cancers [21]. Urban areas have more access to health facilities, especially academic medical centers, which may not be equally available to rural residents. To date, most studies on thyroid cancer have focused on race, gender, socioeconomic status, and stage at diagnosis but not on area of residence (rural vs urban). Nebraska is an agricultural state with a relatively large rural population. In order to receive care in a tertiary care hospital or referral center, persons often have to travel a significant distance to receive medical care, which may pose a barrier to care. In this study, we aimed to identify the effect of rural or urban residence and distance from the treatment center on thyroid cancer stage at diagnosis.

## Methods

### Study Population

We analyzed data collected from 800 thyroid cancer patients enrolled in the Thyroid Tumor and Cancer Collaborative Registry (TCCR), part of the Integrated Cancer Repository for Cancer Research (iCaRe2) bioinformatics and biospecimen registry in the Fred and Pamela Buffett Cancer Center. Adults diagnosed with DTC between 2000 and 2021 were included in the study (n = 800). Enrollment in the registry occurred during the time frame when both the seventh and eighth edition AJCC TNM staging were used, with the eighth edition adopted in 2018. Hence, both seventh and eighth edition AJCC staging was used for analyses of all patients. Patients with anaplastic and medullary thyroid carcinoma and participants with missing data for residence were excluded from the study (n = 32). All participants in the iCaRe2 registry signed informed consent. This study was considered exempt by the University of Nebraska Medical Center (UNMC) institutional review board, since all data obtained from the iCaRe2 registry were de-identified.

### Study Variables

Sociodemographic features (age at diagnosis, gender, race/ethnicity, body mass index [BMI], education, residence [urban vs rural], lifestyle [tobacco/smoking], various clinical and treatment-related data [tumor size, metastasis, lymph node status, procedure type, therapy, and stages of cancer]) were included.

Patients were categorized into rural and urban residence using Rural-Urban Continuum Codes for 2013 (RUCC) and mapping between Federal information Processing Standard (FIPS) and zip codes with the population-based design [22]. RUCC 1-3 was defined as urban and RUCC 4-9 as rural. Access to care was defined by distance between the participant’s residence and the treatment center, categorized as short (≤12.5 miles), intermediate (>12.5 to <50 miles) or long (≥50 miles) distance. This cutoff was used based on previous studies that use the crow-fly variable that measures the distance between the centroid of the patient's zip code or city and the treatment hospital's zip code centroid (as calculated by using the Haversine formula) [23, 24]. The primary outcome was stage at diagnosis. Both seventh and eighth edition AJCC TNM staging for DTC were used for all participants’ analyses. In the seventh edition, 45 years is considered the age cutoff predictive of higher mortality. In the eighth edition, the age cutoff was considered to be 55. When using the seventh edition, subjects were divided into < 45 years and ≥ 45 years at diagnosis. Using the eighth edition, subjects were divided into < 55 and ≥ 55 years at diagnosis. For both seventh and eighth edition, early stage for age < 45 and < 55 respectively was stage I, and late stage was stage II. In those ≥ 45 years and ≥ 55 years at diagnosis for seventh and eighth edition respectively, *early stage* was stage I and II and *late stage* was stage III and IV.

### Statistical Analysis

Differences in patients’ characteristics across geographic categories of rural and urban residence were assessed using a chi-square test. Descriptive statistics (frequencies and percentages) were used for demographic variables, such as age, race, gender, BMI, education, smoking, and clinical characteristics (tumor size, metastasis, surgical procedure, and therapy). A multivariable logistic regression model was used to identify the association of location of residence and distance from hospital with the outcome variable, stage at diagnosis, while adjusting for age at diagnosis and gender. Unadjusted and adjusted odd ratios (ORs) and 95% CIs were reported to assess the association of predictor variables and outcome variables. A *P* value of <.05 was considered statistically significant. Statistical analyses were conducted using the SAS 9.4 version.

## Results

### Participant Demographics

A total of 832 participants were identified. Thirty-two were excluded: 31 had medullary or anaplastic thyroid carcinoma and 1 had a missing RUCC code. The analytic cohort included 800 patients with DTC. Within the cohort, 48.5% were 19 to 44 years of age, 38% were 46-54 years of age, and 13.5% ≥ 65 years (Table 1). The majority (76.2%) were female, non-Hispanic White (91.8%), living with obesity or overweight (76.3%), less than college-educated (50%), and never smokers (63.5%). Most patients had T1 tumors (size 0.1-2.0 cm) (50.3%), 21.7% had T2 (2.1-4 cm), and 27.9% had T3 (> 4 cm). Among them, 64% had no lymph node involvement (N0), 18.7% had central lymph node involvement (N1a), and 16.7% had lateral neck lymph node involvement (N1b). Only 2.8% had distant metastasis. In this cohort, 570 (71.3%) of the participants lived in an urban area, and 230 (28.7%) lived in a rural area. (Table 1). Overall, 224 (28%) lived a short distance, 231 (28.8%) lived an intermediate, and 345 (43.1%) lived a long distance from the treating facility. Breaking down the long-distance group: 155 (45%) participants lived 50 to 100 miles, 129 (37%) lived 100 to 300 miles, and 9 (3%) lived > 300 miles away from the treatment facility. Those living in an urban area comprised all 224 (100%) of the short distance participants, 226 (97.8%) of the intermediate, and 120 (34.7%) of the long-distance participants; in contrast, among those living in a rural area, 5 participants (2.16%) lived an intermediate distance and 225 (65.2%) lived a long distance from the treatment facility (*P* < .0001). Some participants who lived in an urban area were considered as living an intermediate or long distance from a treatment facility if they lived in a community that did not have cancer center treatment facilities and so needed to travel to an alternate urban area to receive care.

**Table 1.** Demographics of entire thyroid cancer cohort

Variables	Urban N (%) = 570 (71.25)	Rural N (%)= 230 (28.7)	Total N (%) = 800 (100)	*P* value*^a^*
Age at diagnosis				**.04**
** **19-44 years	290 (50.8)	98 (42.6)	388 (48.5)	
** **45-64 years	212 (37.1)	92 (40.0)	304 (38.0)	
** **65-95 years	68 (11.9)	40 (17.3)	108 (13.5)	
Gender				.16
** **Female	427 (74.9)	183 (79.5)	610 (76.2)	
** **Male	143 (25.09)	47 (20.43)	190 (23.7)	
Race				
** **Non-Hispanic Whites	506 (89.2)	222 (97.8)	728 (91.8)	
** **Others	61 (10.7)	4 (1.8)	65 (8.1)	
** **Missing	3	4	7	
Education				**.02**
** **Graduate	115 (28.2)	41 (27.8)	156 (28.1)	
** **College graduate	100 (24.5)	21 (14.29)	121 (21.8)	
** **Less than college	192 (47.1)	85 (57.82)	277 (50.0)	
** **Missing	163	83	246 (30.7)	
BMI***^b^***				.18
** **Normal	138 (25.3)	41 (19.16)	179 (23.5)	
** **Overweight	160 (29.3)	65 (30.3)	225 (29.6)	
** **Obese	247 (45.3)	108 (50.4)	355 (46.7)	
** **Missing	15	16	41 (5.1)	
Smoking***^c^***				**.003**
** **Ever smokers	152 (40.3)	36 (26.09)	188 (36.5)	
** **Never smokers	225 (59.6)	102 (73.9)	327 (63.5)	
** **Missing	193	92	285 (35.6)	
Tumor size				.3
** **0.1-2.0** **cm (T1)	284 (52.0)	101 (46.1)	385 (50.3)	
** **2.1-4** **cm (T2)	116 (21.2)	50 (22.8)	166 (21.7)	
** **>4.0** **cm (T3/T4)	146 (26.7)	68 (31.05)	214 (27.9)	
** **Missing	24	11	35 (4.3)	
Lymph nodes				.2
** **Absent (NO)	365 (66.2)	136 (60.1))	501 (64.4)	
** **Central (N1a)	99 (17.9)	47 (20.8)	146 (18.7)	
** **Lateral (N1b)	87 (15.7)	43 (19.0)	130 (16.7)	
** **Missing	33	13	46 (5.7)	
Distant metastasis				.14
** **No	525 (97.7)	208 (95.8)	733 (97.2)	
** **Yes	12 (2.23)	9 (4.15)	21 (2.79)	
** **Missing	193	92		
Therapy type				.13
** **RAI therapy/I-131	298 (53.6)	125 (56.8)	423 (54.5)	
** **No RAI	257 (46.3)	95 (43.1)	352 (45.4)	
** **Missing	15	10	25 (3.1)	
Procedure type				.1
** **Total/near-total thyroidectomy	401 (74.4)	139 (67.1)	540 (72.3)	
** **Lobectomy	120 (22.2)	58 (28.0)	178 (23.8)	
** **Unspecified	18 (3.3)	10 (4.8)	28 (3.7)	
** **Missing	31	23	54 (6.7)	

Excluded underweight BMI due to an extremely low sample. Abbreviations: BMI, body mass index; RAI, radioactive iodine.

^*a*^From the chi-square test.

^*b*^BMI: < 18.5 kg/m^2^ = underweight, 18.5-24.9 kg/m^2^ = normal, 25-29.9 kg/m^2^ = overweight and ≥ 30 kg/m^2^ = obese (CDC, 2017).

^*c*^Smoking status was based on patients who had smoked over 100 cigarettes in their lifetime.

Thyroid cancer was diagnosed at a higher percentage among both urban and rural residents aged 19 to 44 years compared with other age groups (*P* = .04). Rural residents had a higher percentage of participants with less than college education (57.8% vs 47%) (*P* = .02) and more never smokers (73.9% vs 59.6%) (*P* = .003) compared with urban residents. There was no statistically significant difference between the rural and urban groups in terms of tumor size, lymph node metastasis, distant metastasis, or therapy type (Table 1).

Since the AJCC seventh edition prognostic staging considers 45 years as the age cutoff, and the AJCC eighth edition considers 55 years as the age cutoff, we conducted separate analyses for these 2 age groups. In the seventh edition, the younger cohort (age 19-44 years) included 388 patients of whom 290 (74.7%) were urban and 98 (25.26%) were rural residents. The only significant difference between groups was in BMI: 32.3% of urban patients had normal weight vs 18% rural patients (*P* = .04). As expected, the rural patients lived significantly farther from a treating hospital with 98% of the rural group living > 50 miles from the treating center vs 22% of the urban (*P* = .001) patients. There was no statistically significant difference between rural and urban residence or the rest of the variables (Table 2). In the ≥ 45 group, there were 412 participants, with 280 (67.9%) urban and 132 (32.04%) rural residents. The majority (73.7%) were between 45 and 64 years and 26.2% were 65 to 95 years of age at diagnosis. Similar to the younger cohort, significantly more rural participants lived ≥ 50 miles from the treating center (88%) vs 14% in the urban population (*P* < .001). Some of the urban participants lived in larger towns, but still traveled more than 50 miles to receive care. Significantly more rural participants were never smokers (71.4% vs 53.9% in urban) (*P* = .01). Unlike the younger group, there was a difference in tumor size between groups: 34.4% of rural participants presented with tumors > 4 cm, compared with 25.4% in the urban group, and 23.2% of the rural group had tumors of 2.1 to 4 cm, as compared with 19.4% in the urban group (*P* = .05). Hence, stage at presentation was significantly different between groups: 53.1% of the rural group presented as late stage compared with 41.7% of the urban patients (*P* = .034) (Table 3).

**Table 2.** Demographics of AJCC seventh edition cohort: age < 45 years

Variables	Urban N (%) = 290 (74.7)	Rural N (%) = 98 (25.26)	Total N (%) = 388 (100)	*P* value*^a^*
Age at diagnosis				
19-44 years	290 (74.7)	98 (25.26)	388 (100)	
Gender				.17
Female	234 (80.69)	85 (86.73)	319 (82.2)	
Male	56 (19.31)	13 (13.27)	69 (17.7)	
Race				
Non-Hispanic White	262 (90.6)	91 (95.7)	353 (91.9)	
Black/African American	6 (2.08%)	1 (1.05)	7 (1.8)	
Others	20 (6.8)	3 (3.25)	23 (5.9)	
Missing	2	3	5 (1.2)	
Education				**.05**
Graduate	61 (27.9)	10 (13.89)	71 (24.4)	
College graduate	72 (33.3)	27 (37.5)	99 (34.1)	
Less than college	85 (38.9)	35 (48.6)	85 (38.9)	
Missing	72	26	98 (25)	
BMI*^b^*				.**04**
Normal	91 (32.3)	18 (18)	109 (28.9)	
Overweight	74 (26.3)	30 (31.5)	104 (27.6)	
Obese	116 (41.2)	47 (49.4)	163 (43.3)	
Missing	9	3	12	
Smoking*^c^*				.07
Ever smokers	71 (35.3)	16 (23.53)	87 (32.3)	
Never smokers	130 (64.6)	52 (76.4)	182 (67.6)	
Missing	89	30	119 (31)	
Distance to medical facility				.**001**
Short	110 (37.9)	0	110 (28.35)	
Intermediate	117 (40.3)	2	119 (30.6)	
Long	63 (21.7)	96 (97.9)	159 (40.9)	
Tumor size				.9
0.1-2.0 cm (T1)	138 (49.2)	48 (50)	186 (49.4)	
2.1-4 cm (T2)	64 (22.8)	21 (21.8)	85 (22.6)	
>4.0 cm (T3/T4)	78 (27.8)	27 (28.1)	105 (27.9)	
Missing	10	2	12	
Lymph nodes				.6
Absent (No)	169 (60.1)	53 (54.6)	222 (58.7)	
Central (N1a)	64 (22.7)	24 (24.7)	88 (23.2)	
Lateral (N1b)	48 (17.08)	20 (20.6)	68 (17.9)	
Missing	9	1	10	
Distant metastasis				
No	272 (98.9)	96 (98.97)	368 (97.35)	
Yes	3 (1.08)	1 (1.03)	4 (1.06)	
Missing	15	1	16	
AJCC 7 stage				.6
Early: stage (I)	270 (96.43)	92 (94.85)	362 (96)	
Late: stage (II)	10 (3.57)	5 (5.15)	15 (3.9)	
Missing	10	1	11 (2.8)	
Therapy type				.41
RAI therapy/I-131	176 (61.9)	61 (64.8)	237 (62.7)	
No RAI	108 (38.0)	33 (35.1)	141 (37.3)	
Missing	6	4	10	
Procedure type				.72
Total/near-total thyroidectomy	209 (74.6)	66 (70.9)	275 (73.7)	
Lobectomy	64 (22.8)	25 (26.8)	89 (23.8)	
Unspecified	7 (2.5)	2 (2.1)	9 (2.4)	
Missing	10	5	15	

Excluded underweight BMI due to an extremely low sample.

Abbreviations: AJCC, American Joint Committee on Cancer; BMI, body mass index; RAI, radioactive iodine.

^*a*^From the chi-square test.

^*b*^BMI: < 18.5 kg/m^2^ = underweight, 18.5-24.9 kg/m^2^ = normal, 25-29.9 kg/m^2^ = overweight and ≥ 30 kg/m^2^ = obese (CDC, 2017).

^*c*^Smoking status was based on patients who had smoked more than 100 cigarettes in their lifetime.

**Table 3.** Demographics of AJCC seventh edition cohort: age > 45 years

Variables	Urban N (%) = 280 (67.9)	Rural N (%) = 132 (32.04)	Total N (%) = 412 (100)	*P* value*^a^*
Age at diagnosis				.19
45-64 years	212 (75.7)	92 (69.7)	304 (73.7%))	
65-95 years	68 (24.2)	40 (30.3)	108 (26.2)	
Gender				
Female	193 (68.9)	98 (74.2)	291 (70.6)	.26
Male	87 (31.07)	34 (25.7)	121 (29.3)	
Race				
Non-Hispanic White	244 (87.7)	131 (99.9)	375 (91.4)	
Black/African American	24 (8.6)	1 (0.76)	25 (6.10)	
Others	10 (3.6)	0	10 (3.6)	
Missing	2		2	
Education				.3
Graduate	39 (20.6)	11 (14.6)	50 (18.9)	
College graduate	43 (22.7)	14 (18.6)	43 (22.7)	
Less than college	107 (56.6)	50 (66.6)	107 (56.6)	
Missing	91	57	148 (36)	
BMI*^b^*				.8
Normal	47 (17.8)	23 (19.3)	70 (18.2)	
Overweight	86 (32.5)	35 (29.4)	121 (31.5)	
Obese	131 (49.6)	61 (51.2)	192 (50.1)	
Missing	16	13	29	
Smoking*^c^*				**.01**
Ever smokers	81 (46.02)	20 (28.5)	101 (41.06)	
Never smokers	95 (53.9)	50 (71.4)	145 (58.9)	
Missing	104	62	166 (40)	
Distance to medical facility				**<**.**0001**
Short ≤ 12.5 miles	187 (66.7)	0	187 (45.3)	
Intermediate > 12.5 to <50 miles	53 (18.93)	16 (12.1)	69 (16.7)	
Long ≥ 50 miles	40 (14.2)	116 (87.8)	156 (37.86)	
Tumor size				.05
0.1-2.0 cm (T1)	147 (55.06)	53 (42.4)	200 (51.0)	
2.1-4.0 cm (T2)	52 (19.4)	29 (23.2)	81 (20.6)	
>4 cm (T3/T4)	68 (25.4)	43 (34.4)	111 (28.3)	
Missing	13	7	20	
Lymph nodes				.23
Absent (No)	196 (72.5)	83 (64.34)	279 (69.9)	
Central neck (N1a)	35 (12.9)	23 (17.8)	58 (14.5)	
Lateral neck (N1b)	39 (14.4)	23 (17.8)	62 (15.5)	
Missing	10	3	13	
Distant metastasis				.1
No	253 (96.5)	112 (93.3)	365 (95.5)	
Yes	9 (3.4)	8 (6.67)	17 (4.45)	
Missing	18	12	30	
AJCC 7 stage				.**034**
Early: stage (I, II)	156 (58.2)	59 (46.8)	215 (54.57)	
Late: stage (III, IV)	112 (41.7)	67 (53.1)	179 (45.43)	
Missing	12	6	18	
Therapy type				.28
RAI therapy/I-131	122 (45.0)	64 (50.7)	186 (46.8)	
No RAI	149 (54.9)	62 (49.2)	211 (53.1)	
Missing	9	6	15	
Procedure type				.12
Total/near-total thyroidectomy	192 (74.1)	73 (64.0)	265 (71.0)	
Lobectomy	56 (21.6)	33 (28.9)	89 (23.8)	
Unspecified	11 (4.2)	8 (7.0)	19 (5.0)	
Missing	21	18	39	

Excluded underweight BMI due to an extremely low sample.

Abbreviations: AJCC, American Joint Committee on Cancer; BMI, body mass index; RAI, radioactive iodine.

^*a*^From the chi-square test.

^*b*^BMI: < 18.5 kg/m^2^ = underweight, 18.5-24.9 kg/m^2^ = normal, 25-29.9 kg/m^2^ = overweight and ≥30 kg/m^2^ = obese (CDC, 2017).

^*c*^Smoking status was based on patients who had smoked more than 100 cigarettes in their lifetime.

When evaluating using the AJCC eighth edition staging, the younger group (< 55 years) had 549 participants; 73% (403) were urban, and 26.5% (146) participants were rural. The rural cohort had significantly more never smokers (75.2%) vs 62.5% in the urban cohort (*P* = .02). Distance from facility was significantly longer in the rural group, as expected (*P* < .001). There were no other significant differences (Table 4). In contrast, in the older group( ≥ 55 years), distance from facility was significantly different (*P* < .001) and tumor size and AJCC eighth edition staging was also significantly different between groups. Forty percent of the rural cohort presented with T1, 19.7% with T2, and 40.7% with T3 tumor size, compared with 55.7% T1, 17.3% T2, and 27% T3 (*P* = .05). Analysis using the AJCC eighth edition staging showed 83% presenting in early stage among the rural cohort compared with 93% in the urban cohort. Overall, 17% of rural participants presented with late-stage disease, compared with 7% of the urban group (*P* = .17) (Table 5).

**Table 4.** Demographics of AJCC eighth edition cohort: age < 55 years

Variables	Urban N (%) = 403 (73.4)	Rural N (%) = 146 (26.5)	Total N (%) = 549 (100)	*P* value*^a^*
Age at diagnosis				
19-54 years	403 (73.4)	146 (26.5)	549 (100)	
Gender				
Female	317 (78.6)	124 (84.9)	441 (80.3	.10
Male	87 (21.3)	22 (15.07)	108 (19.67)	
Race				
Non-Hispanic White	361 (89.8)	138 (96.5)	499 (91.5)	
Black/African American	15 (3.73)	2 (1.4)	17 (3.12)	
Others	25 (3.6)	2	27 (4.9)	
Missing	2	4	6	
Education				.3
Graduate	82 (27.33)	12 (12.0)	50 (18.9)	
College graduate	97 (32.3)	35 (35.0)	43 (22.7)	
Less than college	121 (40.3)	53 (53.0)	107 (56.6)	
Missing	103	46	149 (27)	
BMI*^b^*				.53
Normal	111 (28.39)	33 (23.57)	144 (27.12)	
Overweight	107 (27.37)	40 (28.57)	147 (27.68)	
Obese	173 (44.25)	67 (47.8)	240 (45.2)	
Missing	12	6	18	
Smoking*^c^*				**.02**
Ever smokers	104 (37.5)	23 (24.7)	127 (34.3)	
Never smokers	173 (62.45)	70 (75.2)	243 (65.6)	
Missing	126	53	179 (33)	
Distance to medical facility				**<.0001**
Short ≤ 12.5 miles	150 (37.22)	0	150 (27.3)	
Intermediate > 12.5 to < 50 miles	163 (40.4)	4 (2.74)	167 (30.4)	
Long ≥ 50 miles	90 (22.3)	142 (97.26)	232 (42.26)	
Tumor size				.91
0.1-2.0 cm (T1)	194 (50.13)	69 (48.5)	263 (49.7)	
2.1-4.0 cm (T2)	89 (23.0)	35 (24.6)	124 (23.4)	
>4 cm (T3/T4)	104 (26.8)	38 (26.7)	142 (26.8)	
Missing	16	4	20	
Lymph nodes				.08
Absent (No)	248 (63.4)	79 (54.4)	327 (61.01)	
Central neck (N1a)	79 (20.2)	42 (28.9)	121 (22.5)	
Lateral neck (N1b)	64 (16.3)	24 (16.5)	88 (16.4)	
Missing	12	1	13	
Distant metastasis				.56
No	376 (98.6)	141 (99.3)	365 (95.5)	
Yes	5 (1.3)	1 (0.7)	17 (4.45)	
Missing	22	4	26	
AJCC 8 stage				.45
Early: stage (I)	385 (98.47)	142 (99.3)	527 (98.6)	
Late: stage (II)	6 (1.53)	1 (0.7)	7 (1.3)	
Missing	12	3	15	
Therapy type				
RAI therapy/I-131	236 (60.5)	91 (64.5)	327 (61.5)	.39
No RAI	154 (39.4)	50 (35.4)	204 (38.4)	
Missing	13	5	18	
Procedure type				.45
Total/near-total thyroidectomy	293 (76.5)	98 (72.0)	391 (75.3)	
Lobectomy	82 (21.4)	36 (26.4)	118 (22.7)	
Unspecified	8 (2.0)	2 (1.4)	10 (1.9)	
Missing	20	10	30	

Excluded underweight BMI due to an extremely low sample.

Abbreviations: AJCC, American Joint Committee on Cancer; BMI, body mass index; RAI, radioactive iodine.

^*a*^From the chi-square test.

^*b*^BMI: < 18.5 kg/m^2^ = underweight, 18.5-24.9 kg/m^2^ = normal, 25-29.9 kg/m^2^ = overweight and ≥30 kg/m^2^ = obese (CDC, 2017).

^*c*^Smoking status was based on patients who had smoked over 100 cigarettes in their lifetime.

**Table 5.** Demographics of AJCC eighth edition cohort: age ≥ 55 years

Variables	Urban N (%) = 167 (66.53)	Rural N (%) = 84 (33.47)	Total N (%) = 251 (100)	*P* value*^a^*
Age at diagnosis				.11
55-74 years	146 (87.43)	67 (79.76)	213 (84.86%)	
75-95 years	21 (12.57)	17 (20.24)	38 (15.14)	
Gender				.48
Female	110 (65.87)	59 (70.24)	169 (67.33)	
Male	57 (34.13)	25 (29.76)	82 (32.67)	
Race				
Non-Hispanic White	145 (87.88)	84 (100)	229 (91.9)	
Black/African American	15 (9.09)	0 (0)	15 (6.02)	
Others	5 (3.03)	0	5 (2.01)	
Missing	2		2	
Education				.79
Graduate	18 (16.82)	9 (19.15)	27 (17.53)	
College graduate	18 (16.82)	6 (12.7)	24 (15.5)	
Less than college	71 (66.36)	32 (68.09)	103 (66.8)	
Missing	60	37	97 (39)	
BMI*^b^*				.36
Normal	27 (17.53)	8 (10.8)	35 (15.35)	
Overweight	53 (34.42)	25 (33.78.)	78 (34.21)	
Obese	74 (48.05)	41 (55.4)	115 (50.44)	
Missing	13	10	23	
Smoking^c^				.03
Ever smokers	48 (48.0)	13 (28.8)	101 (41.06)	
Never smokers	52 (52.0)	32 (71.1)	145 (58.9)	
Missing	67	39	106 (42)	
Distance to medical facility				**<.0001**
Short ≤ 12.5 miles	74 (44.3)	0	7 4 (29.4)	
Intermediate > 12.5 to <50 miles	63 (37.72)	1 (1.19)	64 (25.5)	
Long ≥ 50 miles	30 (17.96)	83 (98.8)	113 (45.02)	
Tumor size				**.05**
0.1-2.0 cm (T1)	87 (55.7)	30 (39.47)	117 (50.43)	
2.1-4.0 cm (T2)	27 (17.31)	15 (19.7)	42 (18.10)	
>4 cm (T3/T4)	42 (26.9)	31 (40.7)	73 (31.47)	
Missing	11	8	10	
Lymph nodes				.33
Absent (No)	117 (73.13)	57 (70.37)	17 4 (72.2)	
Central neck (N1a)	20 (12.5)	7 (8.64)	27 (11.2)	
Lateral neck (N1b)	23 (14.3)	17 (20.9)	40 (16.6)	
Missing	7	3	10	
Distant metastasis				.07
No	149 (95.5)	67 8 (9.3)	216 (93.5)	
Yes	7 (4.49)	8 (10.6)	15 (6.49)	
Missing	11	9	20	
AJCC 8 stage				**.017**
Early: stage (I, II)	146 (92.9)	63 (82.89)	209 (89.7)	
Late: stage (III, IV)	11 (7.01)	13 (17.11)	24 (10.3)	
Missing	10	8	18 (7.1)	
Therapy type				.22
RAI therapy/I-131	57 (35.4)	34 (43.5)	91 (38.0)	
No RAI	104 (64.6)	44 (56.4)	148 (61.9)	
Missing	6	6	12	
Procedure type				.22
Total/near-total thyroidectomy	108 (69.6)	41 (58.5)	149 (66.2)	
Lobectomy	38 (24.5)	36 (26.4)	60 (26.6)	
Unspecified	9 (5.8)	7 (10)	16 (7.1)	
Missing	12	12	26 (10)	

Excluded underweight BMI due to an extremely low sample.

Abbreviations: AJCC, American Joint Committee on Cancer; BMI, body mass index; RAI, radioactive iodine.

^*a*^From the chi-square test.

^*b*^BMI: < 18.5 kg/m^2^ = underweight, 18.5-24.9 kg/m^2^ = normal, 25-29.9 kg/m^2^ = overweight and ≥30kg/m^2^ = obese (CDC, 2017).

^*c*^Smoking status was based on patients who had smoked over 100 cigarettes in their lifetime.

### Impact of Urban/Rural Residence and Distance to Treatment Facility on Cancer Stage Diagnosis

#### Seventh edition AJCC with age cutoff of 45

Both unadjusted (OR 1.5; 95% CI, 1.03-2.42) and age- and gender-adjusted multivariable analyses (OR 1.6; 95% CI, 1.04-2.4) demonstrated higher odds of late stage at diagnosis in rural compared to urban participants in the ≥ 45-year cohort (*P* = .032). Further stratification by age showed that patient age of 65 to 95 years was also significantly associated with late stage at diagnosis as compared with patient ages 45 to 64 years (OR 0.56; 95% CI, 0.35-0.89; *P* = .014) (Table 6). Men were also more likely to present with late stage at diagnosis; (OR 0.53 for women compared to men; 95% CI, 0.34-0.8; *P* = .005) (Table 5). Additionally, participants living a long distance (> 50 miles) had significantly increased odds of late stage at diagnosis (OR 2.0; 95% CI, 1.2-3.2; *P* = .01) after adjusting for age and gender (Table 6 and Table 7).

**Table 6.** Factors associated with late-stage (III/IV) diagnosis in multivariable logistic regression analysis (45 years and older at the time of diagnosis)

Rural/urban category	Significant variables	Unadjusted, OR (95% CI)	*P* value	Adjusted OR (95% CI)	*P* ^a^ value
Urban		Reference			
Rural		1.5 (1.03-2.42)	.034	1.6 (1.04-2.4)	.032
	Sex				
	Male	Reference			
	Female	0.56 (0.36-0.8)	.009	0.53 (0.34-0.8)	.005
	Age at diagnosis				
	65-95 years	Reference			
	45-64 years	0.55 (0.3-0.8)	.010	0.56 (0.35-0.89)	.014

^*a*^Analyses were adjusted for diagnosis age, gender. Abbreviation: OR odds ratio.

**Table 7.** Association between distance from medical facility subgroups and late stage at diagnosis (stage III and IV) in multivariable logistic regression analysis (for 45 years and over at the time of diagnosis)

Distance to center	Significant variables	Unadjusted OR (95% CI)	*P* value	Adjusted *^a^* OR (95% CI)	*P* value
Short ≤ 12.5 miles		Reference			
Long ≥ 50 miles		1.9 (1.2-3.0)	.02	2.0 (1.2-3.2)	.01
	Sex				
	Male	Reference			
	Female	0.56 (0.36-0.8)	.009	0.50 (0.31-0.79)	.0036
	Age at diagnosis				
	65-95	Reference			
	45-64	0.55 (0.3-0.8)	.010	0.58 (0.35-0.95)	.032

Abbreviation: OR odds ratio.

^*a*^Analyses were adjusted for diagnosis age, gender.

#### Eighth edition AJCC with age cutoff of 55

Similar to the seventh edition analysis, patients of rural residence had an increased unadjusted (OR 2.73; 95% CI, 1.16-6.44; *P* = .02) and adjusted (OR 2.57; 95% CI, 1.08-6.14; *P* = .03) odds for late stage at diagnosis. Patients aged 55 to 74 years had a lower risk of late-stage diagnosis than patients of ages 75 to 95 (OR 0.35; 95% CI, 0.13-0.97; *P* = .04) (Table 7). Patients living a long distance from the treatment hospital had significantly increased odds of late-stage diagnosis (OR 4.65; 95% CI, 1.28-16.93; *P* = .075) (Table 8) after adjusting for age and gender. There was no significant difference between male and female participants in the analysis using the eighth edition (Table 8 and Table 9).

**Table 8.** Factors associated with late-stage (III/IV) diagnosis in multivariable logistic regression analysis (55 years and older at the time of diagnosis)

Rural/urban category	Significant variables	Unadjusted, OR (95% CI)	*P* value	Adjusted *^a^*OR (95% CI)	*P* value
Urban		Reference			
Rural		2.73 (1.16-6.44)	.02	2.57 (1.08-6.14)	.03
	Sex				
	Male	Reference			
	Female	0.96 (0.39-2.36)	.93	0.86 (0.34-2.17)	.75
	Age at diagnosis				
	75-95 years	Reference			
	55-74 years	0.33 (0.12-0.87)	.02	0.35 (0.13-0.97)	.04

^*a*^Analyses were adjusted for diagnosis age, gender. Abbreviation: OR odds ratio.

**Table 9.** Association between distance from medical facility subgroups and late stage at diagnosis (stage III and IV) in multivariable logistic regression analysis (for 55 years and over at the time of diagnosis)

Distance to center	Significant variables	Unadjusted OR (95% CI)	*P* value	Adjusted *^a^* OR (95% CI)	*P* value
Short ≤ 12.5 miles		Reference			
Long ≥ 50 miles		4.29 (1.209-15.27)	.0093	4.65 (1.28-16.93)	.0075
	Sex				
	Male	Reference			
	Female	0.96 (0.39-2.36)	.93	0.79 (0.31- 2.019)	.62
	Age at diagnosis				
	75-95	Reference			
	55-74	0.33 (0.125-0.87)	.025	0.29 (0.10-0.82)	.019

Abbreviation: OR, odds ratio.

^*a*^Analyses were adjusted for diagnosis age, gender.

## Discussion

Using the seventh edition AJCC staging, we found age ≥ 45 years, male sex, rural residence, and longer distance from the treatment center were predictors of late stage at diagnosis of DTC. Similarly, using eighth edition staging, age ≥ 55, rural residence, and longer distance from the treatment facility were all independent predictors of higher stage at diagnosis, but male sex was no longer a predictor. This confirms that DTC disparities exist for persons living in rural areas related to access to care. We postulate that patients living in rural areas face greater travel burden including longer distance to the treatment center that requires more time off from work, finding accommodation and prolonged stay away from home, and increased expenses, which delay medical access and consequently affect patient outcomes. A recent study using the National Cancer Database (NCDB) demonstrated that distance from the treatment facility was associated with delays in diagnosis and treatment of thyroid cancer, further supporting our findings [25]. This has also been reported for other cancer types. A meta-analysis reported breast cancer rural patients had 19% higher chances of late-stage diagnosis (OR = 1.19; 95% CI, 1.12-1.27) compared with urban patients [26]. Additionally, Segel at al reported a lower rate of early-stage pancreatic cancer diagnosis among patients living in rural counties [27].

Urban/rural disparities in stage at diagnosis were also reported in an observational study conducted in China among patients with the 5 most common cancers (lung, stomach, colorectum, esophagus, breast cancers) [28]. However, investigating these disparities in the realm of thyroid cancer has been limited, thus highlighting the novelty and importance of our findings. In this study, there was no difference in the proportion of people receiving radioactive iodine (RAI) in the urban vs rural areas, which can appear to be equal access to care. However, since there were more persons diagnosed with higher-stage thyroid cancer in rural areas as compared with urban, this could represent less RAI usage in the rural patients, where arguably there would be increased need in those presenting at higher stages.

Our multivariable analysis revealed that women were less likely to be diagnosed with late-stage thyroid cancer compared with men residing in rural areas. This is in line with previous research, which identified that women with thyroid cancer are diagnosed with smaller tumors (< 4 cm) [5]. Women were found to have frequent healthcare provider visits and receive check-ups more often than men, which may lead to an earlier diagnosis of a smaller tumor [5].

Increasing age is associated with both increased recurrence and mortality of thyroid cancer [29-32]. It has been previously shown that age greater than 55 years was associated with a higher case fatality rate [33]. Additionally, prior studies showed that a well-differentiated thyroid cancer with regional or distant metastasis can have an age-dependent survival rate [33, 34]. Our multivariable subgroup analysis found comparable results, namely that thyroid cancer patients aged 45 to 64 years were 40% less likely to be diagnosed at a late stage compared to those aged 65 to 95 years (OR 0.56; 95% CI, 0.35-0.89), which may translate to lower mortality rates among those younger than 65 years.

Among subjects < 45 years of age, rural residence and distance from the medical facility were not associated with the stage at diagnosis. Also, 96% of these patients were diagnosed at an early stage. These results are consistent with previous studies demonstrating that thyroid cancer patients younger than 45 or 55 years (depending on seventh or eighth edition AJCC staging) can have distinctly different prognosis than those who are older, with age being an important predictor of mortality for DTC. Studies have shown that patients aged 20 to 44 years have a low risk of thyroid cancer recurrence or overall mortality as compared with those ≥ 45 years [34]. The overall favorable prognosis in the < 45-year age group may account for the lack of association between residence and stage at diagnosis in our relatively small study.

A higher percentage of rural patient residents were living with obesity than urban patient residents in our study. Mason et al, in a nationally representative cross-sectional sample studying rural and urban disparities, showed that the odds of obesity were higher in rural compared to urban areas and suggested that obesity and excess body weight were related to increased risk of thyroid cancer [35]. It is possible that the higher prevalence of obesity among rural residents in our cohort may account for more advanced disease at diagnosis. The factors underlying this association need further investigation.

Strengths of the study include using a large thyroid cancer database with participants from both urban and rural areas. Additionally, we used both seventh and eighth edition AJCC TNM staging since staging changed during the duration of enrollment in the registry. The age cutoff impacting stage changed from 45 to 55 in the eighth edition. Studies have shown that 20% of persons with thyroid cancer are downstaged when going from seventh to the eighth edition [36]. To ensure consistency of evaluation between groups, we staged all participants using both seventh and eighth edition and analyzed each group separately.

Limitations of this study include its observational nature and some missing data. Our cohort from the ICaRe2 registry represents a population of thyroid cancer survivors seen at a regional referral center, therefore it is representative of the majority of the referral region including Nebraska, Iowa, Kansas, Missouri, North Dakota, South Dakota. However, it does not capture every person in that region. Also, we did not incorporate education and smoking in the multivariable regression model, to prevent skewed results, since over 30% of these data were missing. At the time of the study, the ICaRe2 registry was only available to English-speaking participants, limiting our ability to evaluate the impact of race/ethnicity on delay in diagnosis. Another limitation is the risk of selection bias in this population. It is unknown whether patients living in rural areas consistently had more advanced disease as a whole or were more willing to travel longer distances if they had advanced disease, and the lower-stage cancers were treated in facilities closer to home. Finally, mortality was not evaluated separately in this study since mortality rates in thyroid cancer are very low. However, AJCC staging has been shown to be a good predictor of disease-specific mortality [37, 38].

## Conclusion

This study demonstrates that residing in rural areas and farther from treatment centers are independently associated with late stage at thyroid cancer diagnosis. Additionally, male sex and age older than 65 years were independently associated with late stage at diagnoses. These findings underscore the importance of identifying barriers to equal access to care as well as ways to implement solutions to close this gap and provide the same access to care for all patients regardless of location of residence. Barriers that need to be further explored include whether medical facilities closer to the rural communities would improve early detection and treatment or impede early referral for specialty care for more advanced disease. Additionally, identification of potential environmental toxins placing rural residents at increased risk of thyroid and other cancers is essential to reduce exposures. Multipronged efforts by clinicians, researchers, medical associations, policymakers, epidemiologists, with the input by community partners are needed to address these disparities and provide a more equitable healthcare landscape.

## Disclosures

S.R., P.F., E.L., and A.G. have no relevant disclosures. A.K. and W.G. are site investigators for a multicenter Siemens Study. W.G. has been a member of the Endocrine Society board, ABIM Endocrinology Board, NCCN, and ATA Guidelines committees, and is an associate editor for *JCEM*. The project described is supported by the National Institute of General Medical Sciences, U54 GM115458, which funds the Great Plains IDeA-CTR Network. The content is solely the responsibility of the authors and does not necessarily represent the official views of the NIH.

## Data Availability

Some or all datasets generated during and/or analyzed during the current study are not publicly available but are available from the corresponding author on reasonable request.

## Abbreviations

AJCC: American Joint Committee on Cancer
BMI: body mass index
DTC: differentiated thyroid carcinoma
iCaRe2: Integrated Cancer Repository for Cancer Research
OR: odds ratio
RAI: radioactive iodine
RUCC: Rural-Urban Continuum Codes
