# Correction to: “Disparities in Thyroid Cancer Diagnosis Based on Residence and Distance From Medical Facility”

**DOI:** 10.1210/jendso/bvag132

**Published:** 2026-06-26

In the above-named article by Regmi S, Farazi PA, Lyden E, Kotwal A, Ganti AK, and Goldner W (J Endo Society. 2024; Volume 8 (Issue 5): 1-12; doi: 10.1210/jendso/bvae033), there was an error in Disclosures.

In the originally published article, the Disclosures section originally stated: “S.R., P.F., E.L., and A.G. have no relevant disclosures. A.K. and W.G. are site investigators for a multicenter Siemens Study. W.G. has been a member of the Endocrine Society board, ABIM Endocrinology Board, NCCN, and ATA Guidelines committees, and is an associate editor for JCEM.” In the corrected article, the Disclosure more completely states: “S.R., P.F., E.L., and A.G. have no relevant disclosures. A.K. and W.G. are site investigators for a multicenter Siemens Study. W.G. has been a member of the Endocrine Society board, ABIM Endocrinology Board, NCCN, and ATA Guidelines committees, and is an associate editor for JCEM. The project described is supported by the National Institute of General Medical Sciences, U54 GM115458, which funds the Great Plains IDeA-CTR Network. The content is solely the responsibility of the authors and does not necessarily represent the official views of the NIH.”

doi: 10.1210/jendso/bvae033

